# Supported Liquid Membranes Based on Bifunctional Ionic Liquids for Selective Recovery of Gallium

**DOI:** 10.3390/membranes12040376

**Published:** 2022-03-30

**Authors:** Haitao Zhou, Yuxi Ye, Yuefei Tan, Kailun Zhu, Xinmin Liu, Hongjing Tian, Qingjie Guo, Lingyun Wang, Shuju Zhao, Yang Liu

**Affiliations:** 1 Key Laboratory of Clean Chemical Processing Engineering of Shandong Province, College of Chemical Engineering, Qingdao University of Science and Technology, Qingdao 266042, China; 17864209795@163.com (H.Z.); yyx3570634@163.com (Y.Y.); tanyf313@163.com (Y.T.); zkl2213312@126.com (K.Z.); lxm220@qust.edu.cn (X.L.); hj_tian@qust.edu.cn (H.T.); qingjie_guo@163.com (Q.G.); 2 School of Metallurgical and Ecological Engineering, University of Science and Technology Beijing, Beijing 100083, China; 3 State Key Laboratory of High-Efficiency Coal Utilization and Green Chemical Engineering, College of Chemistry and Chemical Engineering, Ningxia University, Yinchuan 750021, China; 4 College of Environment and Safety Engineering, Qingdao University of Science and Technology, Qingdao 266042, China; liuyang@qust.edu.cn

**Keywords:** ionic liquid, Bif-ILs, supported liquid membranes, gallium, separation

## Abstract

In this work, separation and recovery of gallium from aqueous solutions was examined using acid-base bifunctional ionic liquids (Bif-ILs) in both solvent extraction and supported liquid membrane (SLM) processes. The influence of a variety of parameters, such as feed acidity, extractant concentration and metal concentration on the solvent extraction behavior were evaluated. The slope method combined with FTIR spectroscopy was utilized to determine possible extraction mechanisms. The SLM containing Bif-ILs demonstrated highly selective facilitated transport of 96.2% Ga(III) from feed to stripping solution after optimization. During the evaluation of the separation performance of SLM for the transport of Ga(III), in the presence of Al(III), Mg(II), Cu(II) and Fe(II), 88.5% Ga(III) could be transported with only 6% Fe(II) and a nil quantity of other metals co-transported. SLM exhibited excellent long-time stability in five repeated transport cycles. Highly selective transport and separation performance was achieved using the SLM containing Bif-ILs, indicating considerable potential for application in Ga(III) recovery.

## 1. Introduction

Due to the high demand for gallium (Ga) resources used in optoelectronics, and particularly for semiconductors consisting of gallium compounded with nitrogen (GaN) or arsenic (GaAs), the utilization of gallium resources has received considerable interest [1,2]. Gallium is generally not found in its own ores, being normally extracted and recovered as a byproduct in bauxite processing and zinc processing [3]. Furthermore, growing demand for gallium makes it crucial to recover it from the inevitably increasing secondary resources, such as waste semiconductors [4]. In hydrometallurgical processes for the recovery of Ga(III) from ores or secondary resources, leaching, adsorption, precipitation, electrolysis, solvent extraction and membrane technologies have been employed [5,6,7].

Among these technologies, solvent extraction was considered to be a preferred technique for extracting and separating Ga(III), due to its high efficiency in extraction and separation [1]. Various molecular extractants and ionic liquids, such as organophosphorus acids [8], neutral extractants [9], amine reagents [10] and quaternary ammonium [11] or quaternary phosphonium salts [12] have been studied for the extraction and separation of Ga(III) from acidic solutions [13]. A stepwise extraction and selective stripping of Ga(III) from Fe(III) and Zn(II) was reported by Zhang et al. [1] using the mixture of tri-n-octylamine and bis(2,4,4-trimethylpentyl) phosphinic acid (Cyanex 272). As a result, 99.7% of gallium was recovered from a strongly acidic sulfate solution. As neutral organophosphorus extractants, Cyanex 923 (mixture of phosphine oxides) and Cyanex 925 (bis(2,4,4-trimethylpentyl) octylphosphine oxide) achieved an efficient separation from Zn(II) and Cu (II) with a 92% recovery efficiency from hydrochloric acid solution [14]. Selective recovery of Ga(III) with an overall extraction percentage of 99% from a leaching solution of zinc refinery residues containing oxalic and sulfuric acid, using a mixture of tri(octyldecyl)amine and tributyl phosphate, was reported by Liu et al. [15]. Nayak and Devi [12] reported the extraction of gallium from hydrochloric acid solution using the ionic liquid Cyphos IL 104 (trihexyl(tetradecyl)phosphonium bis(2,4,4- trimethylpentyl) phosphinate). The selective extraction of Ga(III) (extraction percentage: 99.8%) from Al(III), Cu(II) and Ni(II) was achieved, while the coextraction of Fe(III) and Zn(II) occurred. After the coextraction of Ga(III) and Al(III) from alkaline solution using 7-(4-ethyl-1-methyloctyl)-8-hydroxyquinoline diluted in 1,2,3-triazolium ionic liquid, selective stripping of Ga(III) from Al(III) was obtained by adjusting the stripping solution [2].

A literature survey also indicates that the acid-base coupling bifunctional ionic liquids (Bif-ILs) systems, which show outstanding performance in the extraction and separation of rare earth metals, might be efficient for the selective recovery of Ga(III) from aqueous solutions [16,17]. As for the inner synergistic effect of Bif-ILs, the extraction mechanism was ascribed to the participation of both the cations and the anions in the extraction of metal ions [18]. In solvent extraction processes, stripping and regenerating extractants are important issues in commercialized applications. In the case of novel developed Bif-ILs, although the stripping properties could be improved with the help of adjusting the functional groups, stripping solutions with high concentrations of acidity are still widely used [16]. In the recovery of rare earth metals using Bif-ILs consisting of [trihexyl (tetradecyl) phosphonium][bis(2,4,4-trimethylpentyl)phosphinate], 2 mol/L HNO_3_ solution was used as a stripping solution [19]. Furthermore, large volumes of reagents are needed, and the high viscosity of the ILs generates secondary wastes and increases the energy required in mixing and separating organic and aqueous phases [20].

The technique of using a supported liquid membrane (SLM) impregnated with a tiny quantity of carrier provides an alternative method to exploit ionic liquids with various advantages [21]. SLM is considered effective in improving the separation efficiency of metal ions with a much smaller quantity of ionic liquids, and is able to combine extraction and stripping in one step [22]. Furthermore, ion liquids with high viscosity could ensure relatively high capillary forces, which would prevent the leakage of ILs from the pores of the supporting membrane, which in turn could improve the long term stability of SLMs [23]. Several kinds of ILs, including tri(hexyl)tetradecyl phosphonium chloride, tri-n-octyl methylammonium chloride, 1-butyl-3-methylimidazolium bis[(trifluoromethyl)sulfonyl] imide, etc., have been employed as carriers in SLMs [20,24,25]. As is known, the selectivity and transport mechanism of SLMs depends on the carrier loaded in the membrane. The employment of novel developed Bif-ILs as carriers could provide the possibility for improving the selectivity and stability of SLMs. However, research is very limited in the recent literature in regard to the use of Bif-ILs in SLM for the extraction of metal ions.

Hence, the present work examined the possibility of the selective separation of Ga(III) from hydrochloric acid solution using Bif-ILs in both solvent extraction and supported liquid membrane processes. The effects of various parameters on Ga(III) extraction efficiency were studied, and the extraction mechanism was proposed in both low- and high- acidity conditions. The slope analysis method and spectroscopic characterization were used to analyze the structures of the extracted complexes. The transport of Ga(III) was investigated using one of the optimum Bif-ILs as carrier under different operating conditions, such as feed acidity, initial Ga(III) concentration and stripping solution acidity. The selectivity of the SLM system was evaluated by performing the transport of Ga(III) with other metal ions (Al(III), Mg(II), Cu(II) and Fe(II)) in aqueous solution. The stability and reusability of this SLM system was also examined on a continuous run mode, and the regeneration process was optimized.

## 2. Materials and Methods

### 2.1. Reagents and Synthesis of Bif-ILs

The solutions of individual Ga(III) and mixed solutions of Ga(III) and Al(III), Cu(II), Mg(II) and Fe(II) were prepared by dissolving appropriate amounts of their chloride salts supplied by Sinopharm Chemical Reagent Co., Ltd. (Shanghai, China) distilled water. The commercial extractants, Aliquat336 (95%, Alfa Aesar, Shanghai, China), Cyanex 272 (85%, Cytec Industries Inc., Woodland Park, NJ, USA), PC 88A (95%, Laiyashi Chemical Co., Ltd., Shanghai, China) and D2EHPA (95%, Laiyashi Chemical Co., Ltd., Shanghai, China) were used without any purification.

Binary mixtures of Aliquat 336, D2EHPA, PC88A and Cyanex 272 were prepared by directly mixing them in the desired composition. The Bif-ILs were synthesized by mixing these mixtures and treated by NaHCO_3_ solution according to the method reported in the literature [26,27]. The synthesized ILs are represented as R_4_ND2, R_4_NPC, and R_4_NCy, respectively. The structures of single extractants and bifunctional ionic liquids are presented in Table 1.

### 2.2. Preparation of the SLM

Microporous hydrophilic PVDF membranes (Millipore GVHP 9050, Bedford, MA, USA) with a thickness of 125 μm, porosity of 75% and pore size of 0.22 μm were used as SLM supports. The membrane area available for diffusion was 20 cm^2^. The SLM was prepared according to previously reported procedures [20], by immersing an above-mentioned PVDF membrane in prepared Bif-ILs for at least 24 h at room temperature, then the excess extractants on the surface of the SLMs were erased.

### 2.3. Solvent Extraction and Supported Liquid Membrane Transport Procedure

Equal volumes of extractants and feed solution containing Ga(III) were mixed in a 10 mL centrifugal tube and centrifuged for 15 min (1000 rpm). Thereafter, the raffinate aqueous solution was separated after centrifugation for 6 min and was analyzed for Ga(III) concentration. The concentration of Ga(III) in the loaded organic phase was calculated from mass balance.

**Figure 1:**
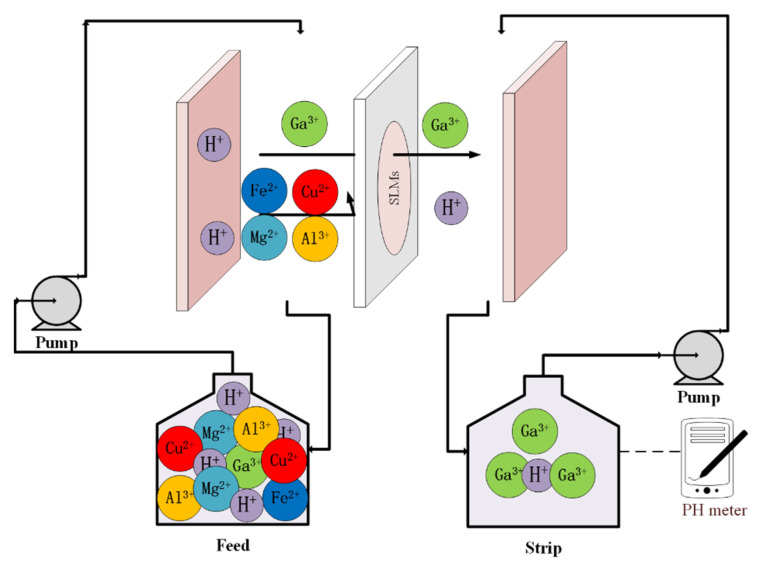
Schematic illustration of the SLM device.

Transport experiments were carried out in a system containing two cylindrical chambers, as shown in Figure 1. The effective membrane area (geometrical membrane area × porosity) of the stack was 15 cm^2^ and the volumes of the feed and stripping solutions were 100 mL. Both feed and stripping solutions were circulated by peristaltic pump (Baoding Qili Precision Pump Corp., Ltd., BT600-01, Baoding, China) with the speed set at 300 rpm. The whole process was thermostated to ambient temperature (T = 25 ± 0.5 °C). Individual experiments were performed three times to ensure the repeatability of the experimental results.

### 2.4. Characterization and Analysis

Both feed and stripping solutions were circulated by peristaltic pumps (Baoding Qili Precision Pump Corp., Ltd., BT600-01, Baoding, China) and kept at a flow rate at 75 mL/min at 300.65 K to avoid concentration-polarization conditions at the interfaces of the membrane and the aqueous solutions. Samples (0.5 mL) of the feed and stripping solutions were periodically taken to analyze the Ga(III) concentration. The extraction percentage (E%) was determined as follows:

(1) $$E\%=\frac{{\left[Ga(\mathrm{III})\right]}_{f,0}-{\left[Ga(\mathrm{III})\right]}_{f,t}}{{\left[Ga(\mathrm{III})\right]}_{f,0}}$$

where [Ga(III)]_f,0_ and [Ga(III)]_f__,t_ are the concentrations of Ga(III) in the feed solution at the start of the experiment (t = 0) and at time t, respectively. Permeability coefficients (P, m/s) were determined by measuring Ga(III) concentration in the feed solution as a function of time, and calculated by using Equation (2):

(2) $$\ln\frac{{\left[Ga(\mathrm{III})\right]}_{f,t}}{{\left[Ga(\mathrm{III})\right]}_{f,0}}=-\frac{A}{V}Pt$$

A is the effective area of prepared membrane (m^2^), while V is the volume of feed solution (m^3^).

In this study, the concentrations of Ga(III) in the feed solution were measured by Atomic Absorption Spectroscopy (Beijing PERSEE Co., Ltd., TAS-986, Beijing, China). The experiments were repeated three times and the average error for measured values was found to be within ±5%. The pH of the stripping solution was measured by a pH meter (WIGGENS Co., Ltd., pH 610, Straubenhard, Germany). Fourier transform infrared spectroscopy (FT-IR) measurements of the organic phases were performed with a Microscopic FT-IR/Raman Spectrometer (Vertex 80 V, Bruker, Germany) in a KBr demountable Cell. The infrared spectra were taken in the range of 4000–550 cm^−1^.In order to study the surface morphology and characteristics of the SLM, scanning electron microscopy (SEM) and energy dispersive X-ray (EDX) (Rigaku Co., Ltd., JMS-6700F; D/MAX-2500/PC, Tokyo, Japan) were used.

## 3. Results

### 3.1. Parameters and Mechanisms of Ga(III) Extraction Using Bif-ILs

**Figure 2:**
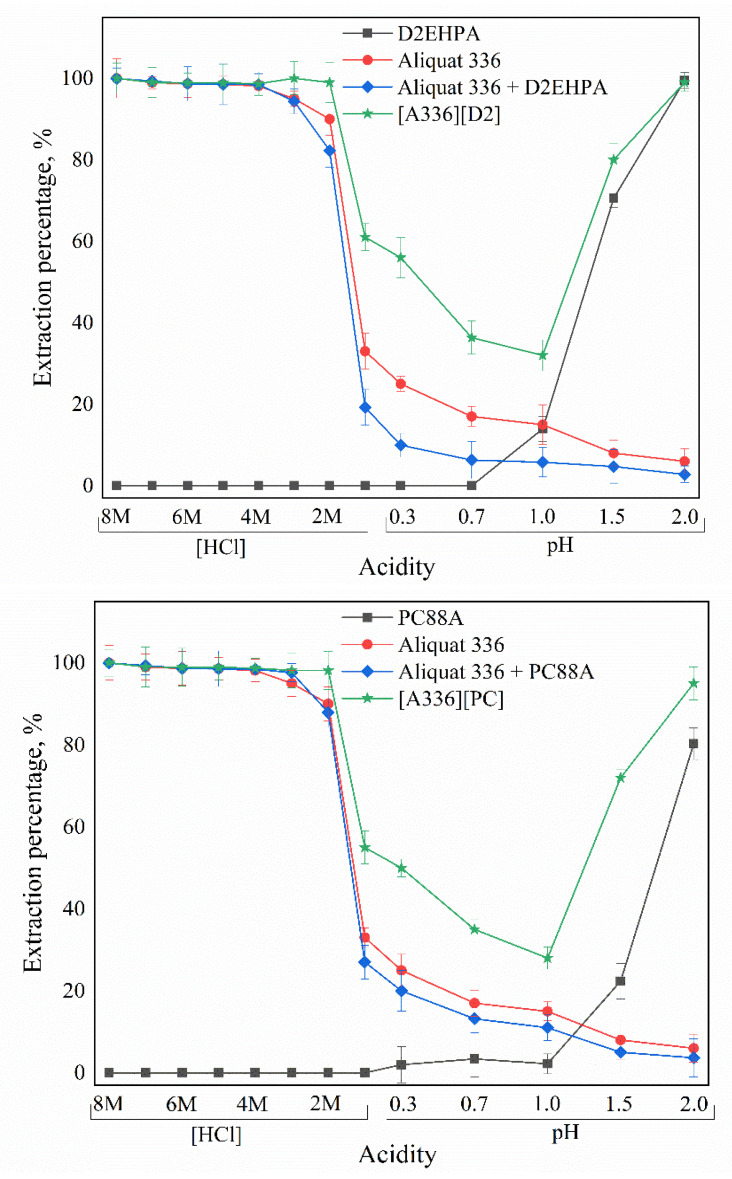

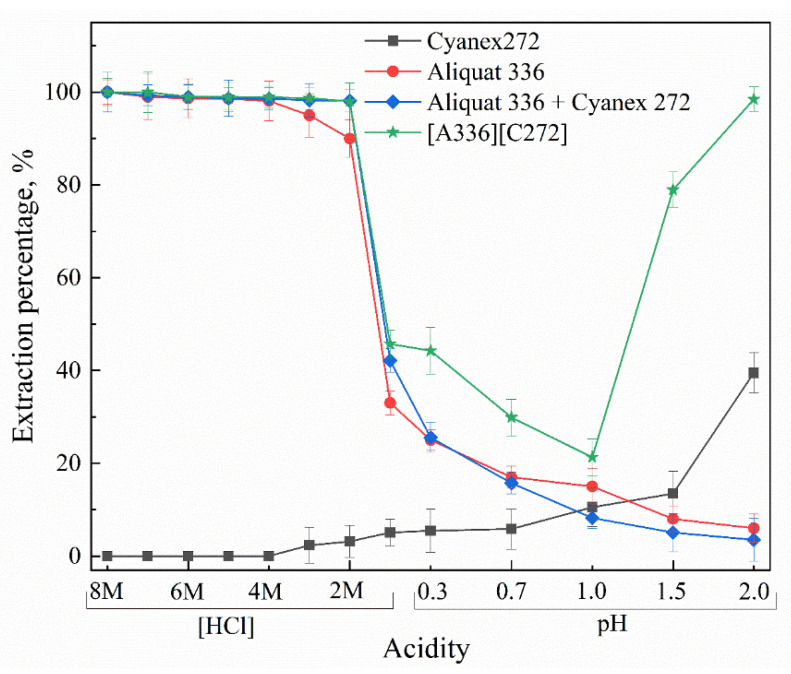
The effect of acidity on the extraction of Ga(III) using acidic extractants, Aliquat 336, binary mixtures and Bif-ILs.

In the solvent-extraction process, the acidity of the feed solution is an important variable for the extraction performance of Ga(III) using Bif-ILs. Therefore, the initial acidity of the feed solution was varied with a wide acidity range (Low acidity: pH range from 0.3 to 2.0; High acidity: 1 M to 8 M HCl) to determine and compare their performance in the extraction of Ga(III) using the acidic extractants, Aliquat 336, binary mixtures and Bif-ILs. The experimental results, shown in Figure 2, show that the extraction percentage of Ga(III) with the different acidic extractants was maintained at a low level at relatively high-acidity conditions. In low-acidity conditions, Ga(III) extraction continuously increased with the increase in the solution pH. Among the three acidic extractants, the extraction percentage of Ga(III) increased with acidity of these extractants, i.e., Cyanex 272 < PC88A < D2EHPA [28]. As illustrated in this figure, the Ga(III)-extraction percentage reached nearly 100% with Aliquat 336, binary mixtures and Bif-ILs at high-acidity conditions, while the extraction percentage decreased with decreasing acidity. For instance, an almost quantitative extraction of Ga(III) at HCl range from 4 M to 8 M HCl decreased sharply to 33.0% and 19.2% when using Aliquat 336 and Aliquat 336 + D2EHPA (pH = 1.0), respectively, and continuously decreased to 6.0% and 2.8%, respectively, at pH 2.0. When mixing acidic extractants with Aliquat 336, an antagonism phenomenon occurred for Ga(III) extraction, indicating the number of functional groups reduced in these systems. Interestingly, the extraction percentage of Ga(III) using Bif-ILs decreased gradually with increasing acidity from pH 2 to pH 1, reached the minimum at pH 1 (0.1M HCl) and then increased again with an increase in HCl concentration from 0.1 M to 8 M. Although the extraction percentage varied with the change in acidity, at each component, Bif-ILs showed better extraction capability than both the individual extractants and binary mixtures. The enhancement of EX% indicates there is a obviously synergistic effect between components in Bif-ILs at both high- and low-acidity conditions, while different extraction mechanisms occur. Both cation exchange and anion exchange mechanisms are involved in the extraction process with aqueous solutions of different acidities, and the Bif-ILs can effectively coordinate with Ga(III) to form a stable extraction complex. Therefore, Ga(III) extraction using Bif-IL in both high- and low-acidity conditions was discussed in detail, and the possible mechanisms were proposed.

To further detect the impact of the Bif-Ils on the extraction of Ga(III) in different acidity conditions (6 M HCl and pH =1.5 as examples), the extraction mechanism was proposed through conventional and effective slope analysis. The slope analysis is a commonly used classical technique for determining the stoichiometry of extracted metal complexes in solvent extraction [10,29].

The extraction of Ga(III) ions in high-acidity solutions toward the Bif-Ils can be written as:

[Ga^3+^]_aq_ + m[R_4_NCy]_org_ + n[Cl^−^]_aq_ + q[H^+^]_aq_ = {[GaCl_n_^−^] [H^+^]_q_[R_4_NCy]_m_}_org_ (3)

where the subscript aq and org indicate aqueous phase and organic phase, respectively. The symbol of m, n and q are the stoichiometries of [R_4_NCy], chloride and hydrogen ion, respectively. Distribution (D_h_) and extraction equilibrium constant (K_ex_, _h_) are defined as Equations (4) and (5), where h indicates the high-acidity condition.

(4) $$D_{h}=\frac{{\left\{\left[{\mathrm{GaCl}}_{n}^{-}\right]{\left[H^{+}\right]}_{q}{\left[R_{4}\mathrm{NCy}\right]}_{m}\right\}}_{\mathrm{org}}}{{\left[{\mathrm{Ga}}^{3+}\right]}_{\mathrm{aq}}}$$

(5) $$K_{\mathrm{ex},\;h}=\frac{{\left\{\left[{\mathrm{GaCl}}_{n}^{-}\right]{\left[H^{+}\right]}_{q}{\left[R_{4}\mathrm{NCy}\right]}_{m}\right\}}_{\mathrm{org}}}{{\left[{\mathrm{Ga}}^{3+}\right]}_{\mathrm{aq}}\times {\left[R_{4}\mathrm{NCy}\right]}_{\mathrm{org}}^{m}\times {\left[\mathrm{Cl}\right]}_{\mathrm{aq}}^{n}\times {\left[H^{+}\right]}_{\mathrm{aq}}^{q}}$$

Modification of Equation (5) leads to Equation (6).

(6) $$K_{\mathrm{ex},\;h}=\frac{D}{{\left[R_{4}\mathrm{NCy}\right]}_{\mathrm{org}}^{m}\times {\left[\mathrm{Cl}\right]}_{\mathrm{aq}}^{n}\times {\left[H^{+}\right]}_{\mathrm{aq}}^{q}}$$

On the basis of Equation (6), taking logarithms:

logD = logK_ex, h_ + mlog[R_4_NCy]_org_ + nlog[Cl^−^]_aq_ + qlog[H^+^]_aq_ (7)

Similarly, the extraction of Ga(III) ions in low-acidity solutions toward the Bif-Ils can be written as:

[Ga^3+^]_aq_ + x[[R_4_NCy]]_org_ + y[Cl^−^]_aq_ + zH_2_O = {[Ga(OH)_z_^−^][Cl_y_^−^] x[R_4_NCy]}_org_ + z[H^+^]_aq_ (8)

where the symbol of x, y and z are the stoichiometries of [R_4_NCy], chloride and hydrogen ion, respectively. Distribution (D_L_) and extraction equilibrium constant (K_ex_, _L_) are defined as Equations (9) and (10), where L indicates the low-acidity condition.

(9) $$D_{L}=\frac{{\left\{\left[\mathrm{Ga}{\left(\mathrm{OH}\right)}_{z}^{-}\right]{\left[{\mathrm{Cl}}^{-}\right]}_{y}{\left[R_{4}\mathrm{NCy}\right]}_{x}\right\}}_{\mathrm{org}}}{{\left[{\mathrm{Ga}}^{3+}\right]}_{\mathrm{aq}}}$$

(10) $$K_{\mathrm{ex},\;L}=\frac{{\left\{\left[\mathrm{Ga}{\left(\mathrm{OH}\right)}_{z}^{-}\right]{\left[{\mathrm{Cl}}^{-}\right]}_{y}{\left[R_{4}\mathrm{NCy}\right]}_{x}\right\}}_{\mathrm{org}}\times {\left[H^{+}\right]}_{\mathrm{aq}}^{z}}{{\left[{\mathrm{Ga}}^{3+}\right]}_{\mathrm{aq}}\times {\left[R_{4}\mathrm{NCy}\right]}_{\mathrm{org}}^{X}\times {\left[\mathrm{Cl}\right]}_{\mathrm{aq}}^{y}}$$

Modification of Equation (10) leads to Equation (11).

(11) $$K_{\mathrm{ex},\;L}=\frac{D\times {\left[H^{+}\right]}_{\mathrm{aq}}^{z}}{{\left[R_{4}\mathrm{NCy}\right]}_{\mathrm{org}}^{x}\times {\left[\mathrm{Cl}\right]}_{\mathrm{aq}}^{y}}$$

On the basis of Equation (11), taking logarithms:

logD = logK_ex, L_ + xlog[R_4_NCy]_org_ + ylog[Cl^−^]_aq_ − zlog[H^+^]_aq_ (12)

i.e.,

logD = logK_ex, L_ + xlog[R_4_NCy]_org_ + ylog[Cl^−^]_aq_ + zpH (13)

**Figure 3:**
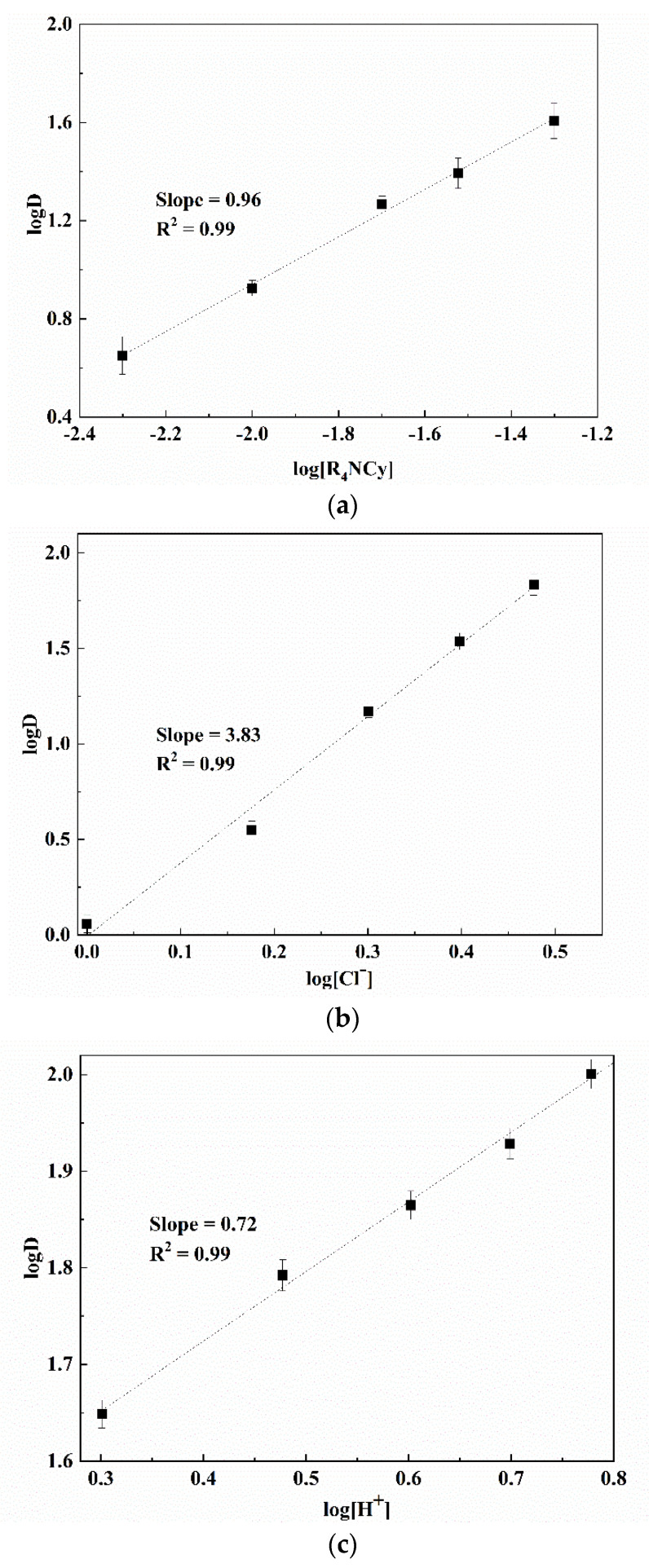
Effects of [R_4_NCy], [Cl^−^] and [H^+^] concentrations on the extraction of Ga(III) at high acidity. (**a**) logD vs. log[R_4_NCy]; (**b**) logD vs. log[Cl^−^]; (**c**) logD vs. log[H^+^].

The log–log relationships between distribution ratio and [R_4_NCy], chloride ion and hydrogen ion concentration were constructed (Figure 3). When the acidity was high, the concentration of [R_4_NCy] was varied, while keeping the concentration of [Cl^−^] and [H^+^] constant. A fitting line was then obtained by plotting the logD against the log [R_4_NCy] as shown in Figure 3a. The slope was 0.96, indicating 1 mol [R_4_NCy] were involved in the extraction of 1 mol Ga(III).The lines of logD against the log[Cl^−^] and log[H^+^] were plotted using a similar procedure. The results obtained in Figure 3b,c indicating the slopes were approximately 4 and 1, respectively. This means that about 4 mol Cl^−^ and 1 mol H^+^ were involved in the extraction of 1 mol Ga(III). Therefore, as a result of the slope analysis and the theory of charge balance, the extraction reaction of Ga(III) from relatively high-acidic solutions using Bif-Ils could be expressed as:

[Ga^3+^]_aq_ + [R_4_NCy]_org_ + [Cl^−^]_aq_ + [H^+^]_aq_ = {[GaCl_4_^−^] [H^+^][R_4_NCy]}_org_ (14)

**Figure 4:**
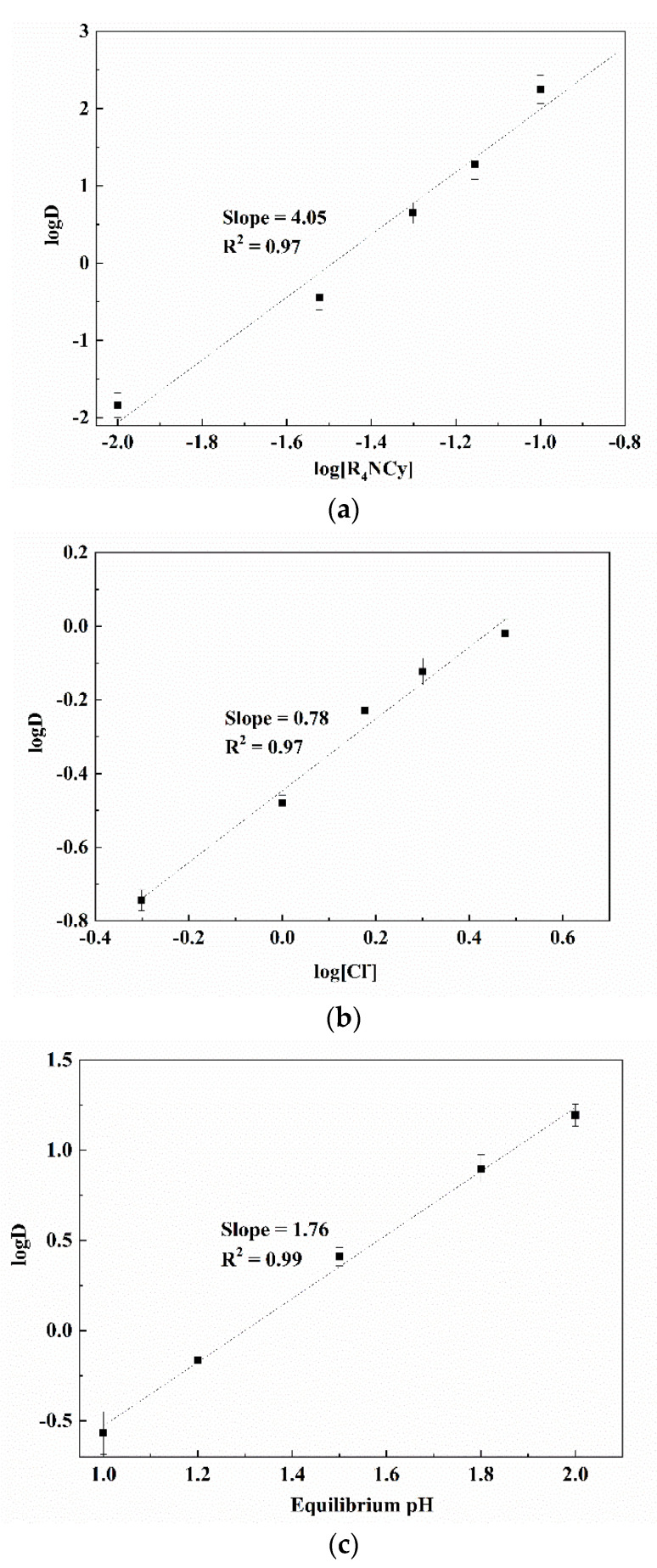
Effects of [R_4_NCy], [Cl^−^] and equilibrium pH on the extraction of Ga(III) at low acidity. (**a**) logD vs. log[R_4_NCy]; (**b**) lgD vs. [Cl^−^] (pH = 1.5); (**c**) logD vs. equilibrium pH.

The linear relationships between logD versus log[R_4_NCy], log[Cl^−^] and equilibrium pH at a low-acid concentration range were obtained with slopes of 4.05, 0.78, and 1.76, respectively (Figure 4). The slope values reveal that the stoichiometries of [R_4_NCy]_org_, [Cl^−^]_aq_ and [H^+^]_aq_ were approximately 4, 1 and 2, respectively.

Accordingly, the extraction mechanism of Ga(III) from relatively low-acidity solutions using [R_4_NCY] system can be represented by the following Equation (4):

[Ga^3+^]_aq_ + 4[[R_4_NCy]]_org_ + [Cl^−^]_aq_ + 2H_2_O = [Ga(OH)_2_^−^Cl^−^ 4[R_4_NCy]]_org_ + 2[H^+^]_aq_ (15)

**Figure 5:**
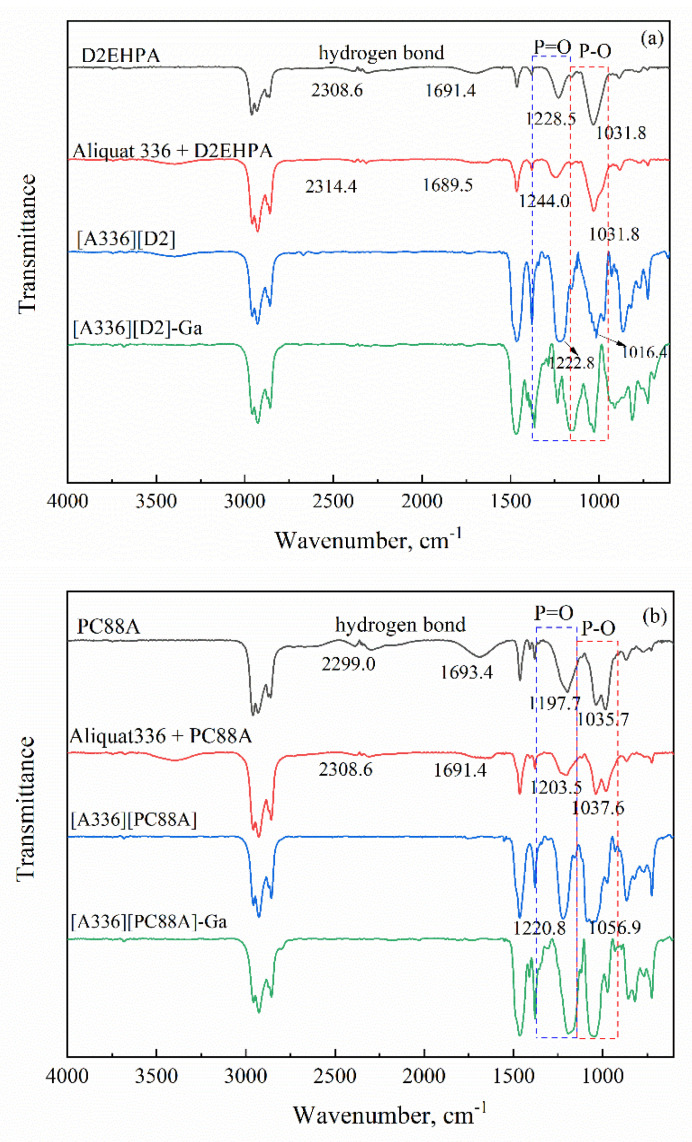

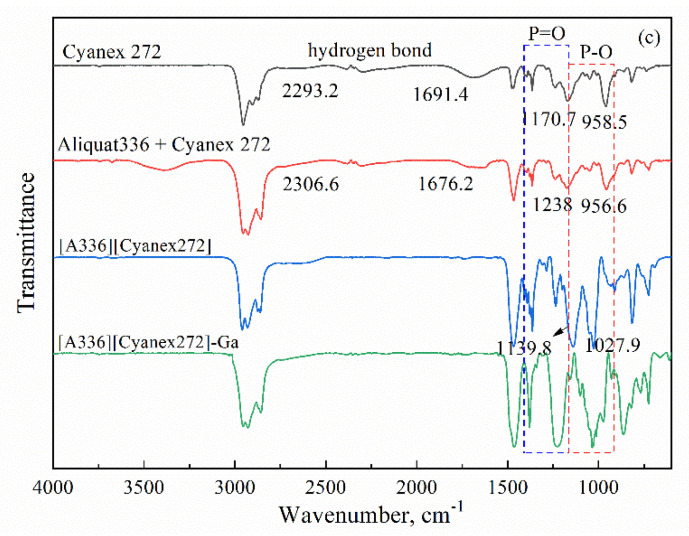
FT-IR spectra of acidic extractants, Aliquat 336, binary mixtures, Bif-ILs and Ga(III)-loaded Bif-ILs. (**a**) D2EHPA based system, (**b**) PC 88A based system, (**c**) Cyanex 272 based system.

The structures of the prepared extractants and the coordination properties of the complexes formed during the extraction process were further determined by FTIR spectroscopy. Figure 5 presents the FTIR spectra of the organic phase containing individual acidic extractants, binary mixtures and Bif-ILs before and after extracting Ga(III). The spectra show that the intensity of stretching vibration of P=O and P-O bonds increased in the Bif-ILs compared to those in individual acidic extractants. Furthermore, the dimeric peaks of hydrogen bands between acidic extractants molecules at around 1690 cm^−1^ and P-OH vibration at around 2310 cm^−1^ disappeared in Bif-ILs, which indicates that hydrogen bonds in the BIf-ILs were completely removed [30]. All of these changes in the characteristic bands in the main types of functional groups of acidic extractants, namely, P=O, P-O, P-O-H and hydrogen bonds, verified the strong ionic interaction between the anions of the acidic extractants and the cations of Aliquat336 in Bif-ILs [27]. A partial shift of the P=O and P-O bands was observed in the Bif-ILs after extracting Ga(III), and was attributed to the conversion of the free ligands to the Ga(III)-ligand complex in the organic phase, indicating that the O atom in the P=O or P-O bond may have interacted with Ga(III) in the aqueous phase.

### 3.2. Mechanistic Investigation of Ga(III) Transport Using Supported Liquid Membrane with Bif-ILs as Carriers

**Figure 6:**
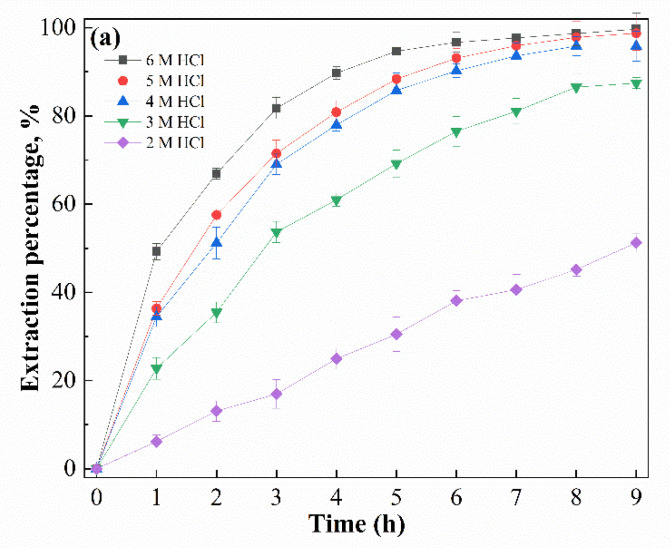

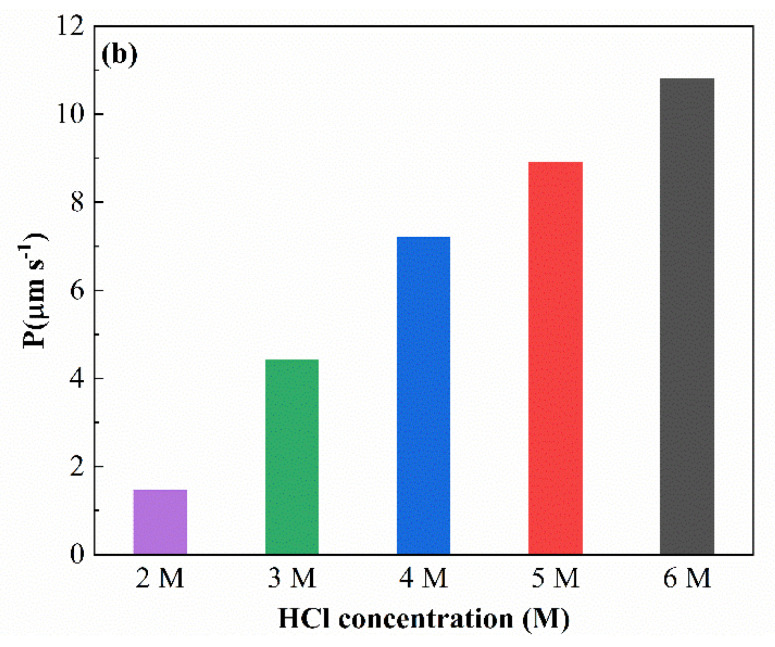
Effect of feed acidity on the transport of Ga(III) by SLMs, (**a**) Extraction percentage, (**b**) permeability coefficient ([Ga(III)]_feed_ = 400 mg/L, Carrier = 0.5 M R_4_NCy, Strip: pH = 1).

It is widely accepted that the acidity levels of both feed and stripping solutions are of great influence in facilitated transport processes using supported liquid membranes [31]. The influence of the initial HCl concentration in the feed solution on the transport of Ga(III) with an SLM containing [R_4_NCy] was assessed, and results are shown in Figure 6. The results show that the extraction percentage elevated sharply from 51.2% to 95.8% as the HCl concentration increased from 2 to 4 M, while the extraction percentage increased smoothly from 98.5% to 99.7% as the HCl concentration increased from 4 to 6 M. Similarly, the permeability coefficient increased from 1.47 to 7.20 μm s^−1^ and from 7.20 to 10.8 μm s^−1^, as the HCl concentration increased. This means that the transport of Ga(III) was accomplished by the interaction between the Bif-ILs and the anionic complex GaCl_4_^−^ formed in high hydrochloride acid solutions at the membrane/feed interface.

**Figure 7:**
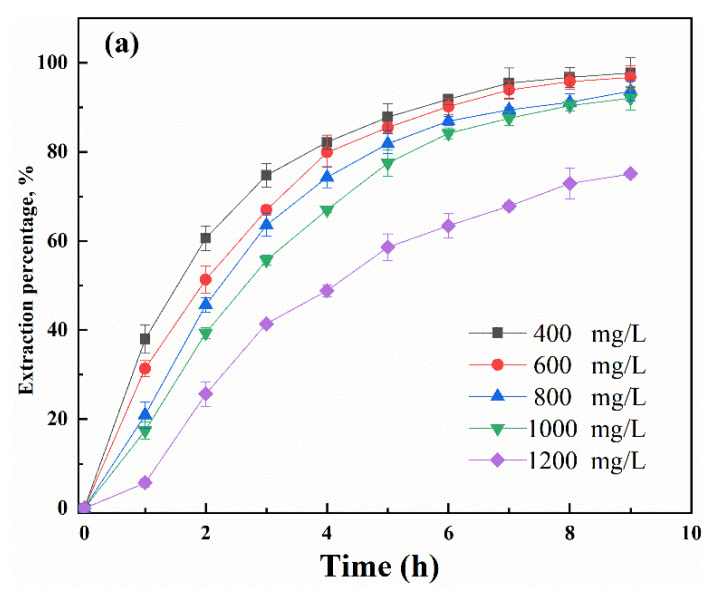

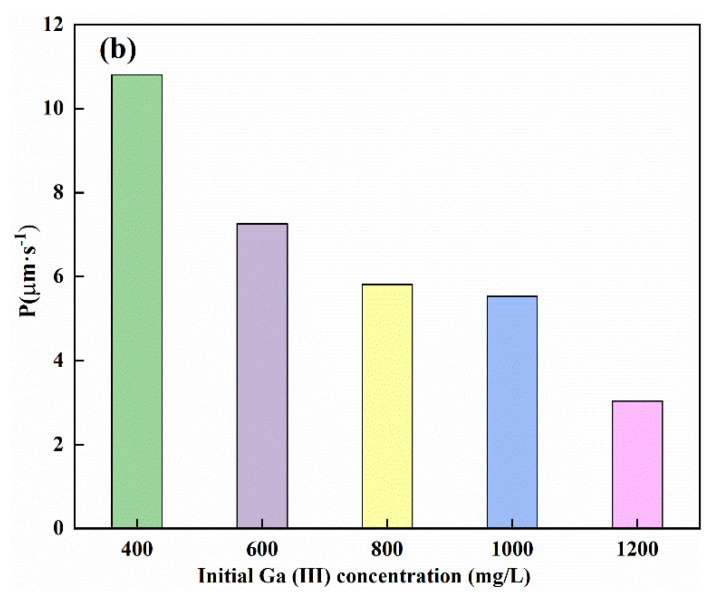
Effect of Ga(III) concentration in feed solution on the transport of Ga(III) by SLMs, (**a**) Extraction percentage, (**b**) permeability coefficient (Acidity: 6 M HCl; Carrier = 0.5 M R_4_NCy, Strip: pH = 1).

The influence of the initial Ga(III) concentration in the feed solution on the SLM transport efficiency was also studied, using feed solutions containing different Ga(III) concentrations ranging from 400 mg/L to 1200 mg/L. It can be seen from Figure 7 that the extraction percentage decreased from 97.8% to 75.1% when the Ga(III) concentration increased from 400 mg/L to 1200 mg/L. The permeability coefficient was decreased from 10.8 to 3.03 μm s^−1^ with the increase in the initial concentration. This was probably caused by the membrane saturation in the presence of a high concentration of metal ion, which lowers the available carriers for the interaction with Ga(III). Therefore, a feed solution containing 400 mg/L Ga(III) was selected for optimization of other experimental parameters.

**Figure 8:**
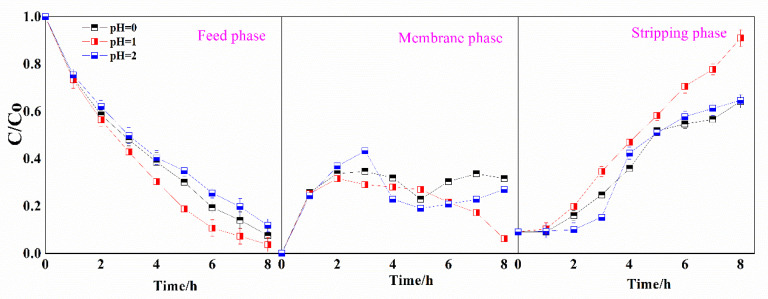
Effect of stripping pH on the transport of Ga(III) by SLMs ([Ga(III)]feed = 400 mg/L, Carrier = 0.5 M R_4_NCy).

The properties of the stripping solution play an important role in both the transport and selectivity in SLM processes. Different pH values ranging between 0 and 2 were examined for their effects on the transport efficiency of Ga(III) through the R_4_NCy-based SLM. As shown in Figure 8, Ga(III) concentration gradually decreased in the feed solution and increased in the stripping solution. Concentrations of 92.4%, 96.2% and 88.2% of Ga(III) in the feed solution could be transported to the stripping solution through SLM with stripping-solution acidity values of pH 0, 1 and 2, respectively. The performance of pH 1 solution as the stripping solution was more efficient compared with that of other stripping solutions of higher or lower pH value. This was coincident with the lowest extractability obtained by Bif-ILs in the solvent extraction process, which indicates that the low interaction between Ga(III) and Bif-ILs in the strip-SLM interface felicitated the transport of Ga(III). The GaCl_4_^−^ complexes were extracted by the Bif-ILs at the feed/SLM interface and then stripped at the SLM/strip phases interface in a continuous process. During the transport, some of the Ga(III) ions were retained in the membrane phase and transported slowly into the stripping solution. It seems that the transport of Ga(III) across the SLM occurred in a diffusion mechanism as the Ga(III) concentration changed in the membrane phase (Figure 8). Furthermore, Ga(III) transport is governed by mass transfer in the membrane, rather than by chemical reactions between Ga(III) species and carrier in the SLM [20].

**Figure 9:**
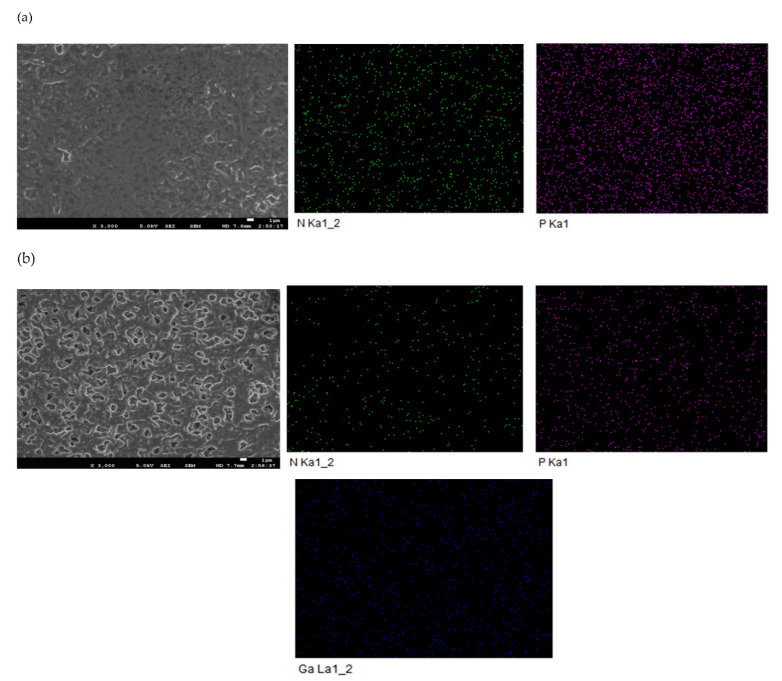
SEM images and EDS elemental mapping of SLM containing R_4_NCy before (**a**) and after (**b**) transport. ([Ga(III)]feed:400 mg/L, Acidity: 6 M HCl, Carrier: Bif-IL (R_4_NCy), Strip: pH = 1; Time: 8 h).

SEM micrographs of SLM containing Bif-IL(R_4_NCy) before and after the transport experiment are displayed in Figure 9. The appearance of P and N belonging to R_4_NCy confirmed that the Bif-IL successfully existed in the membrane and was evenly distributed. The SLM after the experiment showed elevated amounts of Ga(III) loaded on the membrane as a complex with R_4_NCy. Meanwhile, it can be seen from Figure 9 that the quantities of P and N were significantly reduced, which means a small amount of ionic liquid was lost from the membrane pore after the experiment.

### 3.3. SLM Stability

**Figure 10:**
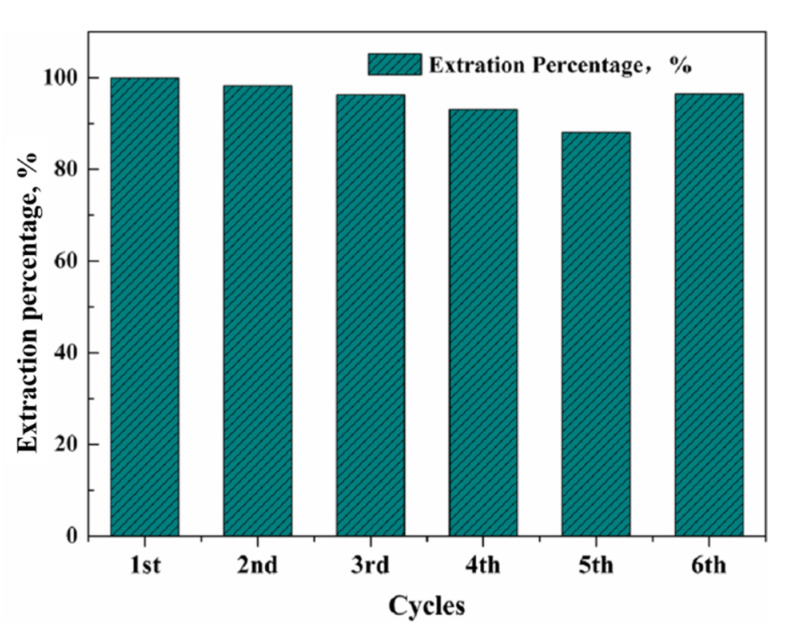
Stability evaluation of the SLM system. ([Ga(III)]feed:400 mg/L, Acidity: 6 M HCl, Carrier: Bif-IL (R_4_NCy), Strip: pH = 1; Time: 8 h; Carriers were impregnated in the 6th cycle).

Although the SLMs performed effectively in metal ion transport, they suffered from lack of stability over time, due to the gradual loss of the carrier in the surrounding aqueous phases, which could affect the permeability and selectivity of SLMs [20,32]. The employment of ionic liquids as carriers is one of the advanced approaches to minimize the instability problem, due to their low stability in aqueous solutions and greater capillary force associated with their high viscosity, which can reduce the leakage of carriers from the pores of supporting membranes [33,34]. To evaluate the stability of the SLM containing R_4_NCy in Ga(III) transport, five successive transport experiments were carried out using the same piece of SLM under optimum conditions, while the membrane was impregnated with fresh carrier at the sixth cycle. Only a slight decrease in the extraction percentage was observed in Figure 10. The corresponding extraction percentage for the five repeated runs was as follows: 99.6%, 98.2%, 95.9%, 92.8% and 88.1%. Although the acidity of the feed solution is very high, it seems that the employed membrane possessed great multiple-use potential. Figure 10 also shows Ga(III) extraction increased to 96.4% after impregnation again at the sixth cycle. The results indicated that the prepared membrane showed satisfactory potential for long-term operation, and could be regenerated again after impregnation.

### 3.4. Selectivity of the Bif-ILs System in Both SX and SLM Processes

**Figure 11:**
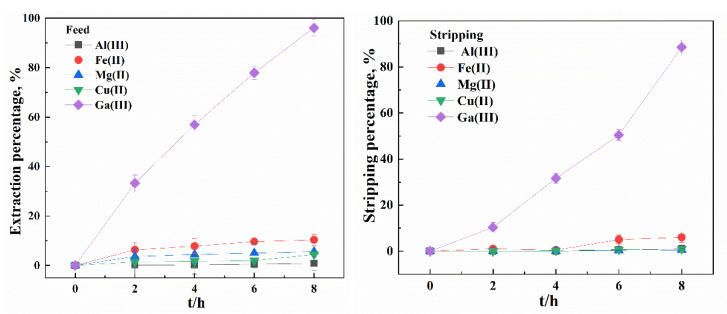
Separation of Ga (III) using R_4_NCy from multicomponent mixed solutions by SLMs ([Ga(III)]/[Al(III)]/[Fe(II)]/[Cu(II)]/[Mg(II)])_feed_ = 400 mg/L, Carrier = 0.5 M R_4_NCy, Strip: pH = 1).

Ga(III) has always co-existed with various kinds of metal ions, depending on the resources of the aqueous solutions, and metal transport in multi-component systems is complicated, due to the diversity of metal ions [31]. Therefore, selectivity behavior of SLM were investigated using synthetic mixed solutions containing Ga(III) in the presence of Al(III), Mg(II), Cu(II), Fe(II) and Fe(III) using R_4_NCy in both SX and SLM processes at optimum conditions. In the SX process, nil extraction of Al(III), Mg(II), Cu(II), Fe(II) (EX%: 3.1%, 0.3%, 3.2%, 0.8%) was found when the Ga(III) extraction percentage was 99.4%. As shown in Figure 11, the SLM system provided preferential transport of Ga(III) in comparison to the other four accompanying metals, with Ga(III) reaching extraction and stripping percentages of 96.1% and 88.5%, respectively. It was also found that although some metal ions could be extracted into the membrane, negligible Al(III), Mg(II) and Cu(II) were transported into the stripping solution, whilst around 6% Fe(II) was transported.

## 4. Conclusions

In this study, the possibility of using novel bifunctional ionic liquids prepared using organophosphorus acids and quaternary ammonium-based ionic liquid in both the solvent extraction and the supported liquid membrane systems for Ga(III) transport was introduced. The extraction system of Bif-ILs demonstrated good performance for Ga(III) extraction in both high- and low-acidity conditions with different extraction mechanisms. The extracted complex compositions were determined to be [H^+^][GaCl_4_^−^][R_4_NCy] and Ga(OH)_2_Cl 4[R_4_NCy], respectively, through the application of a slope analysis method. The study analyzed the effects on SLM transport processes of different transport parameters, such as: feed acidity, initial Ga(III) concentration in feed solution, stripping solution pH and time. Under the optimal transport conditions, 96.2% of Ga(III) in feed solutions containing 6 M HCl could be transported into pH =1 stripping solution. High selectivity was obtained for Ga(III) over other metal ions, including Al(III), Mg(II), Cu(II) and Fe(II), by using R_4_NCy in both solvent extraction and supported liquid membrane systems. After five cycles, the extraction percentage showed only a slight decrease from 99.6% to 88. 1%, which confirmed the stability and reusability of SLM containing R_4_NCy for potential applications.

## Figures and Tables

**Table 1:** Structures of single extractants and bifunctional ionic liquids.

Chemicals (Abbreviations)	Structures
Cyanex 272	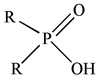
PC88A	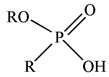
D2EHPA	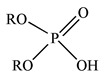
Aliquat 336	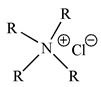
BIf-IL Aliquat336–Cyanex 272 (R_4_NCy)	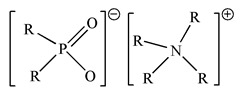
BIf-IL Aliquat336–PC88A (R_4_NPC)	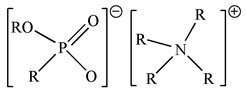
BIf-IL Aliquat336–D2EHPA (R_4_ND2)	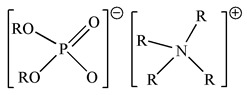

## Data Availability

Not applicable.
